# CD38 Deficiency Alleviates D-Galactose-Induced Myocardial Cell Senescence Through NAD^+^/Sirt1 Signaling Pathway

**DOI:** 10.3389/fphys.2019.01125

**Published:** 2019-09-03

**Authors:** Ling-Fang Wang, Qing Cao, Ke Wen, Yun-Fei Xiao, Ting-Tao Chen, Xiao-Hui Guan, Yu Liu, Li Zuo, Yi-Song Qian, Ke-Yu Deng, Hong-Bo Xin

**Affiliations:** National Engineering Research Center for Bioengineering Drugs and the Technologies, Institute of Translational Medicine, Nanchang University, Nanchang, China

**Keywords:** CD38, D-galactose, oxidative stress, heart senescence, NAD^+^

## Abstract

Our previous research showed that CD38 played vital roles in Ang-II induced hypertrophy and high fat diet induced heart injury. However, the role of CD38 in heart aging is still unknown. In the present study, we reported that CD38 knockdown significantly protected cardiomyocytes from D-galactose (D-gal)-induced cellular senescence. Cellular senescence was evaluated by *β*-galactosidase staining, the expressions of genes closely related to aging including p16 and p21, and the ROS production, MDA content and the expressions of oxidant stress related genes were examined by biochemical analysis, Western blot and QPCR. Our results showed that the expression of CD38 was increased in H9c2 cells after D-gal treatment and the expressions of NAMPT and Sirt1 were downregulated in heart tissue from old mice. CD38 knockdown significantly reduced the number of SA-*β*-gal-positive cells and the expressions of p16 and p21 in H9c2 cells with or without D-gal treatment. The acetylation level of total protein was decreased in CD38 knockdown group, but the expression of Sirt3 was increased in CD38 knockdown group treated with D-gal. In addition, knockdown of CD38 significantly attenuated D-gal induced ROS production, MDA content and NOX4 expression in the cells. Inhibition Sirt1 partially reversed the effects of CD38 knockdown on D-gal induced senescence and oxidative stress. Furthermore, NAD^+^ supplementation reduced D-gal induced cellular senescence, ROS production and MDA content. The expression of SOD2 was increased and the NOX4 expression was decreased in H9c2 cells after NAD^+^ supplementation. Taken together, our results demonstrated that CD38 knockdown alleviated D-gal induced cell senescence and oxidative stress via NAD^+^/Sirt1 signaling pathway.

## Introduction

Senescence is a state of irreversible cellular arrest which is regulated by heredity and environmental factors. The characteristics of aging are involved in various aspects including genomic instability, telomere attrition, mitochondrial dysfunction, ROS accumulation and so on (Lopez-Otin et al., 2013). In developed countries, aging is the biggest risk factor for the leading causes of death, and the incidence of cardiac disease increases dramatically with age (Kaeberlein, 2013). The data from American Heart Association in 2014 showed that the incidence of cardiovascular diseases for 60 to 79 years of age is >70% and for >80 years of age is >80% in America (Go et al., 2014). The alternations associated with cardiac aging mainly included four levels: functional, structural, cellular and molecular (Steenman and Lande, 2017). Mitochondrial dysfunction and oxidative stress had been known as important factors in heart aging. Emerging evidences revealed that mitochondrial production of ROS was significantly increased in the heart with advanced age (Judge et al., 2005; Lesnefsky et al., 2016; Martin-Fernandez and Gredilla, 2016). Therefore, it is important to find efficient methods to inhibit oxidative stress and improve mitochondrial function, then delaying senescence of heart.

Nicotinamide dinucleotide (NAD^+^) is a key cofactor for maintaining the cellular energy metabolism. Several studies reported that the content of NAD^+^ was declined during aging and senescence (Mouchiroud et al., 2013; Verdin, 2015; Zhu et al., 2015). Accumulative evidences revealed that NAD^+^ supplementation or other approaches that can restore NAD^+^ levels were highly protective during aging (Belenky et al., 2007; Mouchiroud et al., 2013; Mills et al., 2016; Katsyuba et al., 2018). CD38 is a major hydrolase for degradation of NAD^+^, and expressed in many tissues. It has been founded that the content of NAD^+^ in heart and brain tissues was increased significantly in CD38 deficiency mice than wild-type mice (Aksoy et al., 2006). Recently, a study showed that CD38 gradually increased in tissues of liver, muscle and adipose during aging, and the involvement of CD38 in aging might be in part due to down-regulated Sirt3 activity (Camacho-Pereira et al., 2016). Besides, it was reported that several metabolic features of aging were mitigated with a potent and specific inhibitor of CD38 by reversing tissue NAD^+^ decline (Tarrago et al., 2018). Sirtuins were class III histone deacetylases, which used NAD^+^ as substrate. A number of evidences indicated that Sirtuin family had beneficial effects against age-associated damage and oxidative stress (Ho et al., 2009; Qiu et al., 2010; Someya et al., 2010; Tasselli et al., 2017). Our previous study showed that CD38 deficiency had protective roles in many cardiac diseases including ischemia/reperfusion injury, cardiac hypertrophy induced by Ang-II and lipid overload-induced heart injury (Guan et al., 2016; Guan et al., 2017; Wang et al., 2018a). However, whether CD38 affects heart aging and the mechanisms remains unknown.

In the current study, in order to test the roles of CD38 in heart aging, D-galactose (D-gal) was used to establish an aging cell model with CD38 knockdown H9c2 stable cell line. Our results showed that D-gal promoted cardiomyocytes senescence and increased ROS production, and the expression of CD38 was up-regulated in senescent cardiomyocytes, while the expressions of NAMPT and Sirt1 were down-regulated in old mice heart. CD38 knockdown could attenuate myocardial cell aging and oxidative stress induced by D-gal, while Sirt1 specific inhibitor EX-527 reversed the effects of CD38 deficiency on senescence and oxidative stress. In addition, our results revealed that the effects could be rescued by NAD^+^, suggesting that the NAD^+^/Sirt1 signaling may play important roles in myocardial cells aging induced by D-gal.

## Materials and Methods

### Chemicals and Antibodies

D-Galactose (Cat. No: G5388), NAD^+^ (Cat. No: N5755) and EX-527 (Cat. No: E7034) were purchased from Sigma-Aldrich (St. Louis, MO). Antibodies against p16, p21, NAMPT, NOX4, and GAPDH were purchased from Abcam. Acetylated-Lysine, LC3B, Sirt3, and SOD2 antibodies were purchased from CST. Antibody against Sirt1 was purchased from Millipore. CD38 antibody was from R&D Systems.

### Animals

Male C57BL/6 mice were used in our experiment. The mice were fed with standard diet chow and housed in an animal room with a 12-h: 12-h light/dark cycle under controlled environment (22 ± 3°C, 50–60% relative humidity). Two to 3 month-old mice were used as the young group and 11- to 12-month-old mice as old group. All animals were treated according to the “guidelines for the care and use of experimental animals” of Nanchang University. All experimental procedures were approved by the Ethics Committee of Nanchang University, and the experiments were conducted according to the approved guidelines.

### Cell Culture and Treatment

H9c2 cells (ATCC) were regularly cultured in high-glucose DMEM containing 10% fetal bovine serum, 100 U/ml penicillin and 100 mg/ml streptomycin at 37°C in a humidified incubator under 5% CO2. The CD38 knockdown H9c2 cell line was prepared as previously described in reference (Guan et al., 2016). When the cells were about 80% confluence, the cells were treated with D-Galactose or combined with Sirt1 specific inhibitor EX-527 (25 μM) for 48 h, then the cells were harvested for further experiments. When it comes to NAD^+^ treatment, the H9c2 cells were treated with D-Galactose for 24 h, then cells were incubated with NAD^+^ (1.0 mM) for another 24 h before analysis. H9C2 stable cell line with knockdown of CD38 was prepared in our laboratory as previously described (Guan et al., 2016). The NC was defined as siRNA control H9C2 stable cells which were transfected with scramble control siRNA and selected with 1 μg/mL puromycin.

### SA-*β*-Gal Staining

To evaluate myocardial cell senescence induced by D-galactose, senescence-associated *β*-galactosidase (SA-*β*-gal) staining was performed according to the instruction. Briefly, after D-galactose treated, the cells were washed with phosphate buffered saline (PBS) and fixed with 4% paraformaldehyde for 15 min at room temperature. After washing three times with PBS, the cells were incubated in SA-*β*-gal staining solution overnight at 37°C. Then the staining cells were observed under light microscope, and the senescent cells were blue color. The experiment was repeated three times in each group.

### ROS Detection

The content of ROS production in cells was examined using H2DCF-DA (Sigma-Aldrich). Briefly, the cells underwent corresponding treatment, then were washed with PBS. About 1 × 10^6^ cells were collected and incubated with 10 μM H2DCF-DA for 30 min at 37°C with an atmosphere of 5% CO_2_ away from light. Then the cells were washed with PBS and the fluorescence was detected with automatic microplate reader at wavelengths of 488 nm (excitation) and 520 nm (emission).

### Measurement of Peroxidation Levels

The content of MDA in cells was detected using MDA Assay Kit (Dojindo, Mashiki-machi, Japan) according to the manufacturer’s instructions. Protein concentration in lysates was determined by BCA Protein Assay Kit (Pierce). All experiments were performed at least three times.

### Total RNA Extraction and Real-Time RT-PCR

Total RNA from myocardial cells and heart tissue was isolated using Trizol reagent (Invitrogen) according to the manufacturer’s protocol. The concentration of RNA was measured by Nano Drop 2000. Then the RNA was experienced reverse transcription with Takara high capacity cDNA synthesis kit. Relative expression of mRNAs was measured by real-time PCR with SYBR Premix Ex Taq^TM^ II (Takara) in the ABI-ViiA7 PCR machine. All PCR reactions were performed in triplicate. The following primer pairs were used: Rat CD38, 5-CTGCCAGGATAACTACCGACCT-3 (Forward) and 5-CTTTCCCGACAGTGTTGCTTCT-3 (Reverse); GAPDH, 5-AGCCAAAAGGGTCATCATCT-3 (Forward) and 5-GGGGCCATCCACAGTCTTCT-3 (Reverse); Mouse p16, 5- CGGGGACATCAAGACATCGT-3 (Forward) and 5- GCCGGATTTAGCTCTGCTCT-3 (Reverse); Mouse NAMPT, 5-TCGGTTCTGGTGGCGCTTTGCTAC-3 (Forward) and 5- AAGTTCCCCGCTGGTGTCCTATGT-3 (Reverse).

### Western Blot Analysis

Total protein was prepared with RIPA Lysis Buffer (Thermo Fisher). Then the lysates were centrifuged at 12,000 rpm for 15 min at 4°C. The protein concentration was determined by BCA Protein Assay Kit (Pierce). A total of 30 μg of each protein sample was resolved by SDS-PAGE and transferred to PVDF membranes and then the membranes were blocked with 5% non-fat milk for 1 h and then incubated overnight at 4°C with indicated antibodies. The membranes were then washed three times with TBST and then incubated with horseradish peroxidase- (HRP-) conjugated anti-rabbit or anti-mouse secondary antibodies. At last, the proteins were detected with enhanced chemiluminescence (ECL) (Thermo Fisher). GAPDH was used as the internal control.

### Statistical Analysis

All experiments were performed at least three times. Data are presented as means ± SE. And statistical analysis was performed with SPSS (Statistical Package for the Social Sciences) 19.0 software using Student’s *t*-test. Statistical significance was set at ^∗^*p* < 0.05, ^∗∗^*p* < 0.01, ^∗∗∗^*p* < 0.001.

## Results

### CD38 Expression and the Senescence Were Significantly Increased in D-Gal Induced Myocardial Cells

**FIGURE 1:**
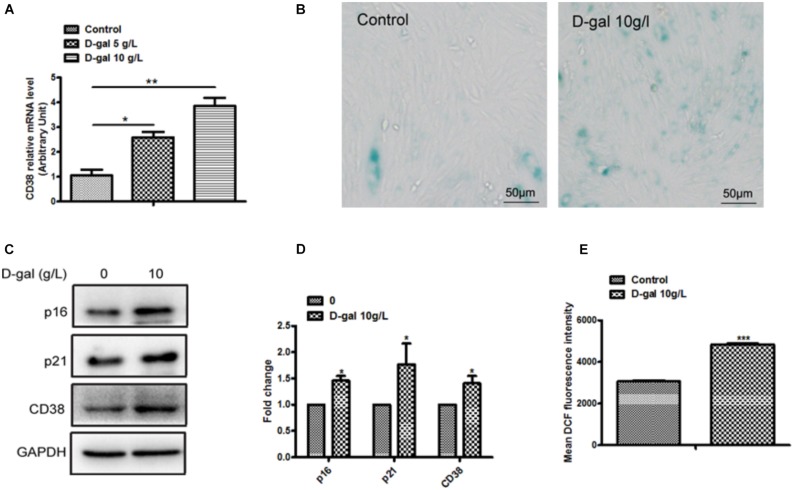
Myocardial cells senescence and ROS production were increased by D-gal. **(A)** The expression of CD38 by real-time PCR analysis in H9c2 cells treated with different concentrations of D-gal. **(B)** SA-*β*-gal staining in H9c2 cells treated with D-gal (10 g/L). **(C)** The images of senescence marker p16, p21 and CD38 protein by Western blot analysis in H9c2 cells treated with D-gal (10 g/L). **(D)** Quantitative analysis of p16, p2,1 and CD38 protein level from western blot bands. **(E)** The mean fluorescence intensities of ROS production were quantitatively analyzed in H9c2 cells treated with D-gal. Data are shown as mean ± SEM, ^∗^*p* < 0.05, ^∗∗^*p* < 0.01 and ^∗∗∗^*p* < 0.001, *n* = 3 per group.

To explore the potential role of CD38 in heart aging, we first examined the CD38 expression in H9c2 cells treated with D-gal. The results showed that the expression of CD38 was markedly increased in H9c2 cells after D-gal treatment (Figures 1A,C). Moreover, to investigate the effects of D-galactose on myocardial cell senescence, we used SA-*β*-Gal staining to evaluate cellular senescence. The staining results showed that 10 g/L D-gal remarkably increased SA-*β*-gal-positive cells compared with control group (Figure 1B). Furthermore, we found the expressions of senescence marker including p16 and p21 were significantly increased in the H9c2 cells treated with D-gal (Figures 1C,D). These results suggested that 10 g/L D-gal could promote cellular senescence, and the CD38 expression was increased in D-gal treated cells. Besides, we also found that the content of ROS production has a significant increase in H9c2 treated with D-gal (Figure 1E). All the results indicated that D-gal also increased oxidative stress of myocardial cells.

### Cellular Senescence Was Increased and Sirt1 Signaling Pathway Was Inhibited in Heart Tissue of Old Mice

**FIGURE 2:**
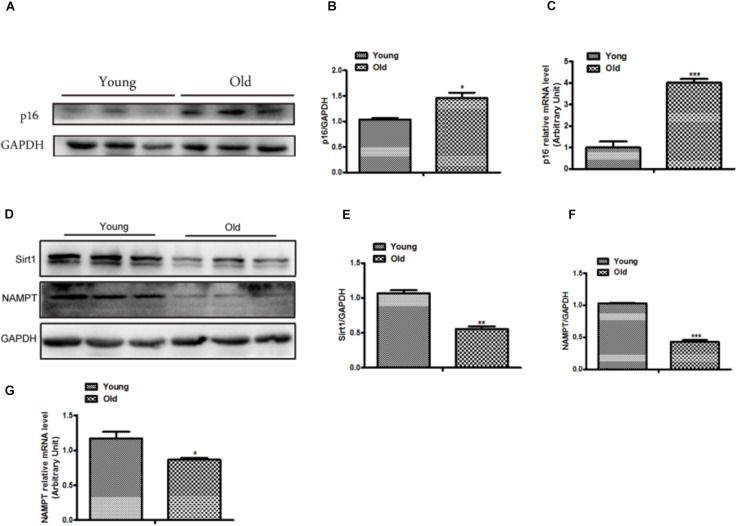
The expression of p16 was increased and the expressions of Sirt1 and NAMPT were decreased in heart tissue from old mice. The western blot image **(A)** and the quantitative analysis **(B)** of p16 protein were determined in heart tissue from old and young mice. **(C)** The mRNA expression of p16 was determined by qPCR in in heart tissue from old and young mice. The Western blot images **(D)** and the quantitative analysis **(E,F)** of the Sirt1 and NMAPT proteins were determined in heart tissue from old and young mice. **(G)** The mRNA expression of NAMPT was determined by qPCR. The data are expressed as the mean ± SEM. from three independent experiments. ^∗^*p* < 0.05, ^∗∗^*p* < 0.01 and ^∗∗∗^*p* < 0.001.

To further confirm whether cellular senescence was increased in heart tissue of old mice, we evaluated the senescence in heart tissue from mice approximately one year old. The results suggested that the expression of p16 has a remarkable increase in old group compared with young group (Figures 2A,B), and the mRNA level of p16 was consistent with the protein (Figure 2C). Moreover, the proteins related to Sirt1 signaling pathway were altered between old and young mice, as shown in Figures 2D–G, the expressions of Sirt1 and NAMPT which has been considered as the pivotal rate-limiting enzyme in the mammalian NAD^+^ salvage pathway, were significantly decreased in old mice. Moreover, we also found the expression of Sirt1 was decreased in D-gal induced cellular senescence (Supplementary Figure S1B). Taken together, our results demonstrated that cellular senescence was increased and the Sirt1-mediated signaling pathway was inhibited in old mice.

### CD38 Knockdown Attenuated D-Gal Induced Myocardial Cells Senescence

**FIGURE 3:**
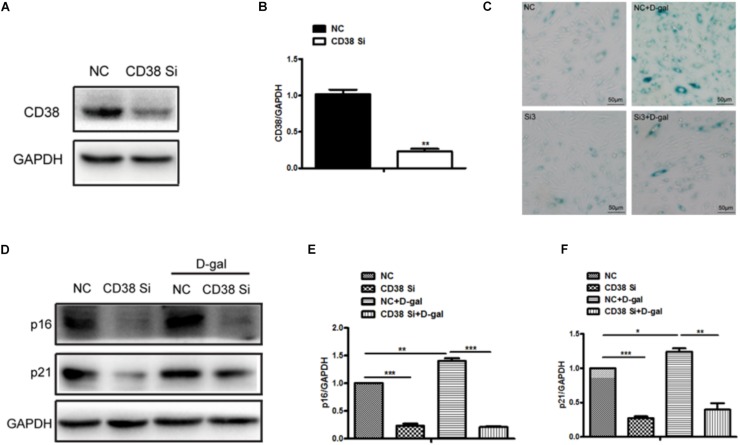
CD38 knockdown decreased D-gal induced myocardial cells senescence. **(A)** The CD38 protein expression was determined by western blot **(A)** and the quantitative analysis **(B)** in the CD38 knockdown stable H9c2 cell lines. **(C)** SA-*β*-gal staining in CD38 knockdown H9c2 cells treated with or without with D-gal (10 g/L). **(D)** The Western blot images **(D)** and the quantitative analysis **(E,F)** of senescence marker p16 and p21 protein were determined in CD38 knockdown H9c2 cells treated with or without with D-gal (10 g/L). The data are expressed as the mean ± SEM. from three independent experiments. ^∗^*p* < 0.05, ^∗∗^*p* < 0.01 and ^∗∗∗^*p* < 0.001.

In order to further elucidate the roles of CD38 in myocardial senescence induced by D-gal *in vitro*, we examined the effects of CD38 on D-gal-induced senescence using CD38 knockdown H9c2 stable cell lines. The interference efficiency of CD38 was approximately 90% by western blotting analysis (Figures 3A,B). Furthermore, the SA-gal-positive cells were markedly decreased in CD38 knockdown group compared with control group after D-gal treatment using SA-*β*-Gal staining (Figure 3C). And in CD38 knockdown H9c2 cells, the senescence was aggravated after treated with D-gal combined with Sirt1 specific inhibitor EX-527 (Supplementary Figure S1C). In addition, we found the expressions of senescence marker p16 and p21 were reduced in CD38 knockdown H9c2 cells with or without D-gal stimulation (Figures 3D–F). These results indicated that CD38 knockdown improved D-gal induced myocardial cells senescence and inhibition of Sirt1 partially reversed the effects of CD38 deficiency on senescence *in vitro*.

### CD38 Knockdown Decreased D-Gal Induced Myocardial Cells Oxidative Stress

**FIGURE 4:**
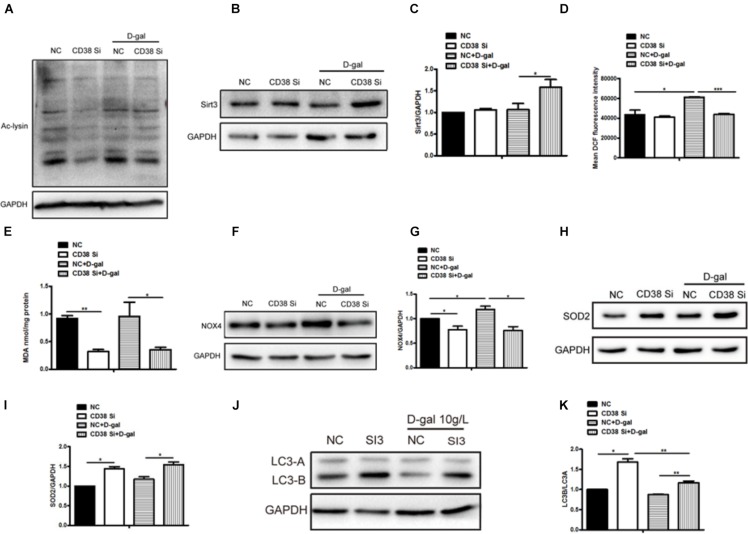
CD38 knockdown decreased D-gal induced oxidative stress in cardiomyocytes. **(A)** The protein acetylation level of CD38 knockdown H9c2 cells treated with or without with D-gal (10 g/L) was analyzed by western blotting with antibody against acetylated-lysine. The western blot images **(B)** and quantitative analysis **(C)** of the protein level of Sirt3 were determined in CD38 knockdown H9c2 cells. **(D)** The mean fluorescence intensities of ROS production were quantitatively analyzed in CD38 knockdown H9c2 cells treated with or without with D-gal. **(E)** The content of MDA was measured in CD38 knockdown H9c2 cells treated with or without with D-gal. The western blot images **(F)** and quantitative analysis **(G)** of the protein level of the oxidative stress related gene NOX4 were determined in CD38 knockdown H9c2 cells. The western blot images **(H)** and quantitative analysis **(I)** of the protein level of the anti-oxidative stress related gene SOD2 were determined in CD38 knockdown H9c2 cells. The western blot images **(J)** and quantitative analysis **(K)** of the protein level of the autophagy associated gene LC3 were determined in CD38 knockdown H9c2 cells. The data are expressed as the mean ± SEM. from three independent experiments. ^∗^*p* < 0.05, ^∗∗^*p* < 0.01 and ^∗∗∗^*p* < 0.001.

Aging is often accompanied by an increase in oxygen free radicals. Besides senescence, we also found that oxidative stress was increased after D-gal stimulation. Then we further explore the roles of CD38 in D-gal induced oxidative stress. The results showed that the level of total protein acetylation increased in H9c2 cells under D-gal treatment, while CD38 knockdown reduced it with or without D-gal stimulation (Figure 4A and Supplementary Figure S1E), indicating that the sirtuin activity might be increased. Beisdes, the expression of Sirt3 which was mainly located in mitochondria was increased in CD38 knockdown cells after D-gal treatment (Figures 4B,C). Moreover, the ROS production was increased by D-gal, but decreased by CD38 knockdown in cells treated with D-gal (Figure 4D), and EX-527 reversed the effects of CD38 deficiency on oxidative stress (Supplementary Figure S1D). In addition, we also detected the content of MDA in D-gal induced CD38 knockdown H9c2 cells, and the indicator could reflect the degree of oxidation damage. The results showed that the level of MDA in cells declined in the group of CD38 knockdown with or without D-gal treatment (Figure 4E). Besides, the expression of NOX4, which was associated with ROS production, was increased upon treatment with D-gal, but decreased by CD38 knockdown (Figures 4F,G). And we found the expression of antioxidant gene SOD2 was upregulated in CD38 knockdown group with or without D-gal stimulation (Figures 4H,I). Moreover, the expression of LC3B/LC3A was significantly increased in CD38 knockdown cells, suggesting that the autophagy might be increased (Figures 4J,K). The results above indicated that CD38 knockdown decreased D-gal induced oxidative stress, and Sirt1 was responsible for D-gal induced senescence and oxidative stress.

### NAD^+^ Partially Rescued D-Gal Induced Cellular Senescence and Oxidative Stress

**FIGURE 5:**
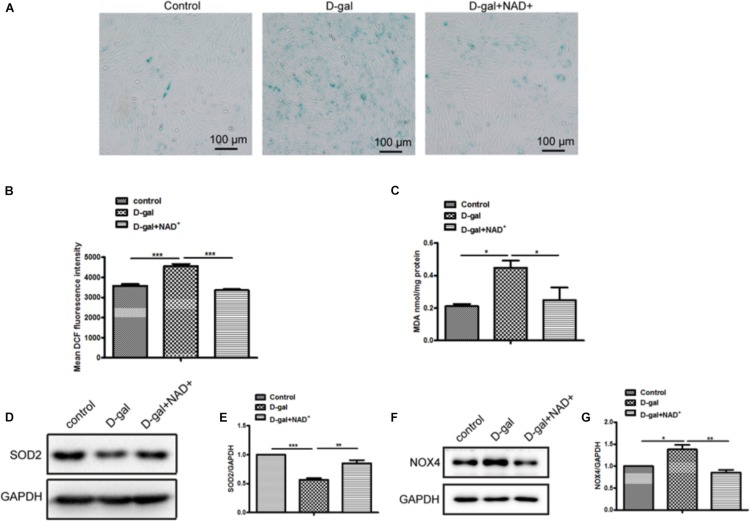
NAD^+^ supplement improved D-gal induced senescence and oxidative stress in cardiomyocytes. **(A)** SA-*β*-gal staining was performed in H9c2 cells treated with D-gal (10 g/L) or combined with NAD^+^ (1.0 mM). **(B)** The mean fluorescence intensities of ROS production were quantitatively analyzed in H9c2 cells treated with D-gal (10 g/L) or combined with NAD^+^ (1.0 mM). **(C)** The content of MDA was measured in H9c2 cells. The western blot images **(D)** and quantitative analysis **(E)** of the protein level of the oxidative stress related gene SOD2 were determined in H9c2 cells. The western blot images **(F)** and quantitative analysis **(G)** of the protein level of the oxidative stress related gene NOX4 were determined in H9c2 cells. The data are expressed as the mean ± SEM. from three independent experiments. ^∗^*p* < 0.05, ^∗∗^*p* < 0.01 and ^∗∗∗^*p* < 0.001.

The roles of NAD/Sirt signaling in D-gal induced cellular senescence were examined in H9c2 cells after supplement NAD^+^ under D-gal treatment. The SA-*β*-Gal staining showed that the SA-*β*-gal-positive cells were reduced in cells treated with D-gal plus NAD^+^ compared with D-gal alone (Figure 5A). Meanwhile, the ROS production was markedly decreased by supplement of NAD^+^ in H9c2 cells treated with D-gal (Figure 5B). Besides that, the content of MDA was decreased in cells treatment with NAD^+^ (Figure 5C). Furthermore, the results showed that NAD^+^ increased the expression of SOD2 but decreased the expression of NOX4 in H9c2 cells compared with D-gal treatment group alone (Figures 5D–G). Taken together, these results demonstrated that NAD^+^ supplement could partially rescue D-gal induced cellular senescence and oxidative stress.

## Discussion

Accumulating studies reported that the amount of people who are over 65 years old will be double from 12% in 2010 to 22% in 2040 (Heidenreich et al., 2011). Cardiovascular disease (CVD) is the main reason causing death in older people and in turn age-associated changes in endothelial and cardiac cells may enhance the risk of CVD development (Olivieri et al., 2013). The main characteristics of cardiac aging include cardiac cell death, hypertrophy and fibrosis. The senescent cells usually expressed senescence-associated *β*-galactosidase and p16^INK4A^, so SA-*β*-gal staining and the expression of p16 are most commonly used to evaluate aging. D-galactose is widely used for preparing mouse aging model (Li et al., 2016; Liao et al., 2016), and it has been reported that D-gal significantly decreased rat heart function (Chang et al., 2016). In addition, subcutaneous injection of galactose could impair cognitive performance in rodents (Sadigh-Eteghad et al., 2017), so D-gal was also used to examine brain aging. In the present study, we used D-gal to induce senescence in cardiomyocytes. The results showed that 10 g/L D-gal increased myocardial cell senescence which was reflected in increased SA-*β*-gal staining and the expressions of senescence marker p16 and p21. Meanwhile, the ROS production was also increased in H9c2 cells treated with D-gal. More importantly, the expression of CD38 was upregulated in H9c2 cells at mRNA and protein level after treated with D-gal, suggesting that CD38 may play a role in D-gal-induced cardiomyocytes senescence.

CD38 is an important hydrolase of NAD^+^. The intracellular level of NAD^+^ was associated with aging and related diseases (Zhang et al., 2016; Li et al., 2017). NAD^+^ concentrations were decreased in animals during aging and senescence (Verdin, 2015). Our previous study also demonstrated that CD38 played vital roles in adipogenesis and high fat induced oxidative stress (Wang et al., 2018a, b). But the role of CD38 in heart aging was unknown. In this study, we did examine the expression of CD38 in heart tissue from 2 to 3 month-old mice (young) and 11 to 12-month-old mice, unfortunately we did not observe the increase of CD38 expression in the heart tissue from old mice (11 to 12-month-old) compared to young mice (Supplementary Figure S1A). The possible reason might be due to that the 11 to 12-month mice were too young for aging research since it has been reported that CD38 was up-regulated in several tissues such as liver, adipose tissue, spleen and muscle at least in 24 month-old mice (Camacho-Pereira et al., 2016). But we showed that the expression of Sirt1, a key molecule involved in NAD^+^ metabolism, was significantly down-regulated in old mice, and the expression of NAMPT, a rate-limiting enzyme in NAD^+^ salvage pathway, was also decreased in heart tissue from old mice. These results further demonstrated the important roles of NAD^+^ metabolism in heart aging.

To further explore the role of CD38 in myocardial cell senescence, a CD38 knockdown stable H9c2 cell line was used in the study. The interference efficiency was about 80 percent. Our results showed that CD38 knockdown markedly decreased the SA-*β*-gal-positive cells and the expressions of senescence marker p16 and p21 compared with control group. P16^INK4A^ is a selective inhibitor of cyclin D-dependent CDK4 and CDK6 and commonly used as a marker for evaluating senescence. It was reported that p16 was markedly increased in almost all rodent tissues with advancing age (Krishnamurthy et al., 2004). However, since p16 also has apparent limitations as a biomarker of senescence *in vivo* the combination of SA-*β*-gal staining and p16 is more reliable in evaluating aging.

Oxidative stress is closely related to many diseases, including aging. Cardiac aging is often accompanied by accumulating damage of mitochondrial and increasing level of reactive oxygen species (ROS) production in myocardiocytes and heart tissues (Kong et al., 2014). Many papers have presented that D-gal can induce oxidative stress in aging and other models (Li et al., 2016; Qiu et al., 2017). In the current study, our results showed the intracellular protein level of acetylation, the production of ROS, the content of MDA, and the expression of NADPH Oxidase 4 which was an indicator of oxidative stress significantly declined in CD38 knockdown group treated with D-gal, indicating that CD38 knockdown efficiently inhibited oxidative stress induced by D-gal. And the ROS production was aggravated after treated with D-gal combined with Sirt1 specific inhibitor EX-527. In addition, study showed that autophagy played pivotal roles in the heart during the aging process (Shirakabe et al., 2016). Our results suggested autophagy level was significantly increased in the group of CD38 knockdown, manifested as an increased expression of LC3B/LC3A. However, the relationship between the CD38-mediated decreased autophagy and increased oxidative stress needs to be further study. Taken together, these findings indicated that CD38 knockdown inhibited the oxidative induced by D-gal and increased autophagy.

NAD^+^ concentrations were decreased in animals during aging (Gomes et al., 2013), suggesting that NAD^+^ supplementation might exert protective effects during aging (Zhang et al., 2016). In this study, NAD^+^ supplementation reduced H9c2 cell senescence, the production of ROS and MDA content when treated with D-gal. On the contrary, the expression of antioxidant gene SOD2 was increased, but the expression of NOX4 was decreased after NAD^+^ supplementation. These results indicated that NAD^+^ supplementation could reduce senescence and oxidative stress induced by D-gal *in vitro*.

## Conclusion

Our results demonstrated that NAD^+^/Sirt signaling played important roles in heart aging. CD38 knockdown alleviated D-gal induced cell senescence and oxidative stress, and NAD^+^ supplementation had the similar effects with CD38 knockdown. Obviously, this study will provide new insights in elucidating the mechanism of heart aging and finding the therapeutic targets for delaying or preventing heart aging.

## Data Availability

All datasets for this study are included in the manuscript and the Supplementary Files.

## Ethics Statement

The animal study was reviewed and approved by the Ethics Committee of Nanchang University.

## Author Contributions

L-FW and QC performed the experiments, participated in the design of the study, and carried out the animal model. KW, Y-FX, and X-HG participated in the data analysis. H-BX and K-YD conceived the study and participated in the design and coordination of the study. L-FW drafted the manuscript. H-BX revised the manuscript. All authors read and approved the final version of the manuscript.

## Conflict of Interest Statement

The authors declare that the research was conducted in the absence of any commercial or financial relationships that could be construed as a potential conflict of interest.

## Supplementary Material

FIGURE S1 Sirt1 was responsible for D-gal induced senescence and oxidative stress and reversed the effects of CD38 knockdown on it. **(A)** The western blot image of CD38 protein were determined in heart tissue from 1 month and 12 month old mice. **(B)** The western blot image of Sirt1 protein in H9c2 cells treated with D-gal (10 g/L). **(C)** SA-*β*-gal staining in CD38 knockdown H9c2 cells treated with or without with D-gal (10 g/L) or combined with Sirt1 specific inhibitor EX-527 (25 μM). **(D)** The mean fluorescence intensities of ROS production were quantitatively analyzed in H9c2 cells treated with D-gal (10 g/L) or combined with EX-527 (25 μM). **(E)** The protein acetylation level of CD38 knockdown H9c2 cells treated with or without with D-gal (10 g/L) was analyzed by western blotting with antibody against acetylated-lysine. Data are shown as mean ± SEM, ^∗^*p* < 0.05, ^∗∗^*p* < 0.01 and ^∗∗∗^*p* < 0.001, *n* = 3 per group.
