# Supplementary figures and images for: CD38 Deficiency Alleviates D-Galactose-Induced Myocardial Cell Senescence Through NAD+/Sirt1 Signaling Pathway
Source: Front Physiol. 2019 Sep 3;10:1125. doi: 10.3389/fphys.2019.01125 (PMC6735286; doi:10.3389/fphys.2019.01125)

**Figure S1**

**A**

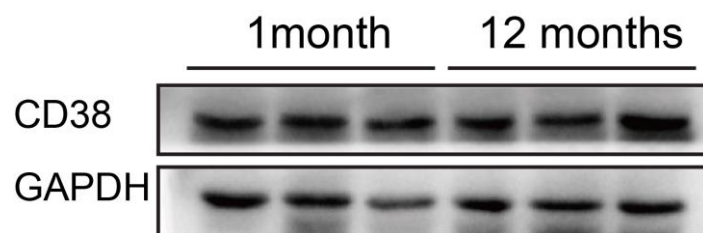

**B**

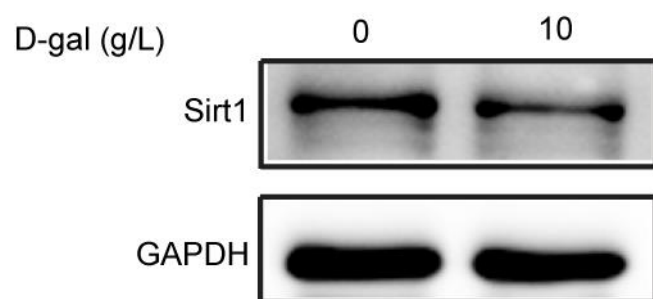

**C**

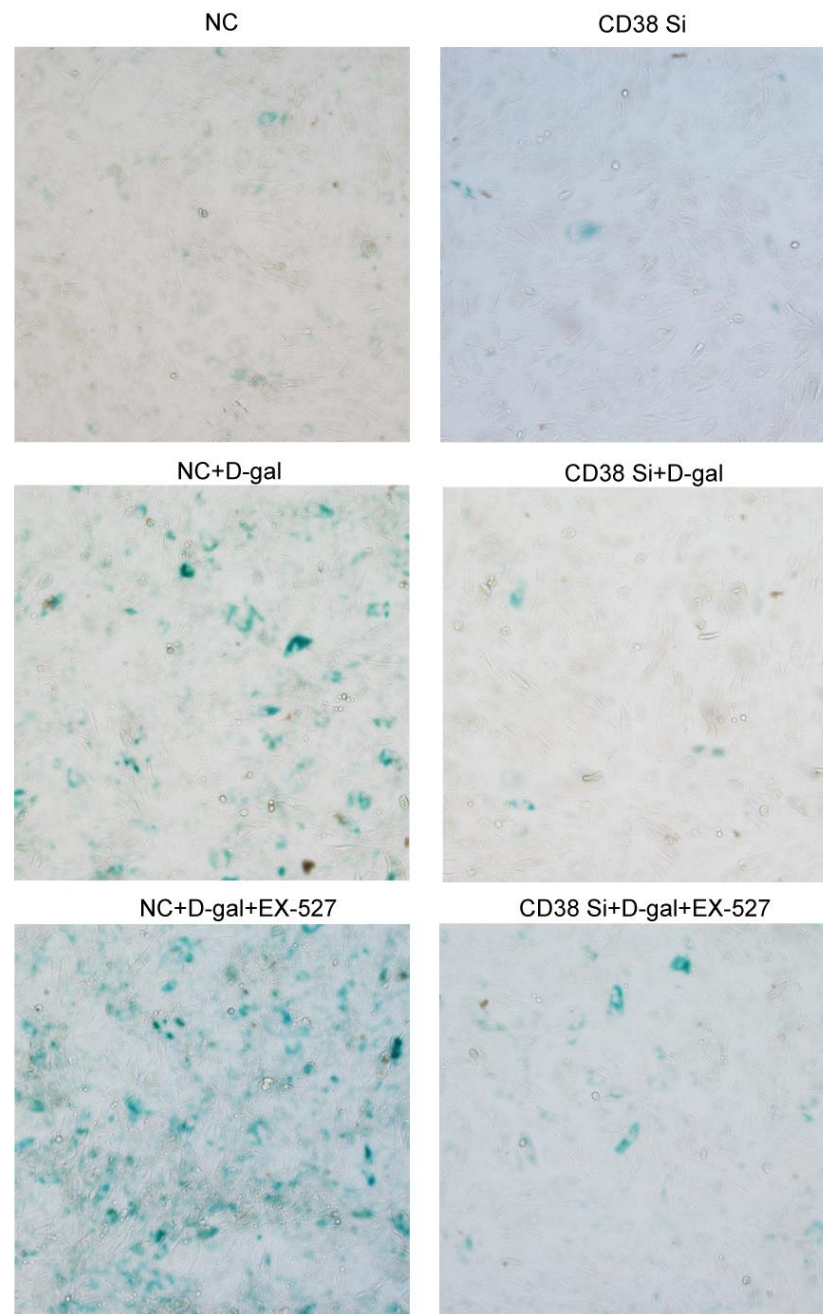

D

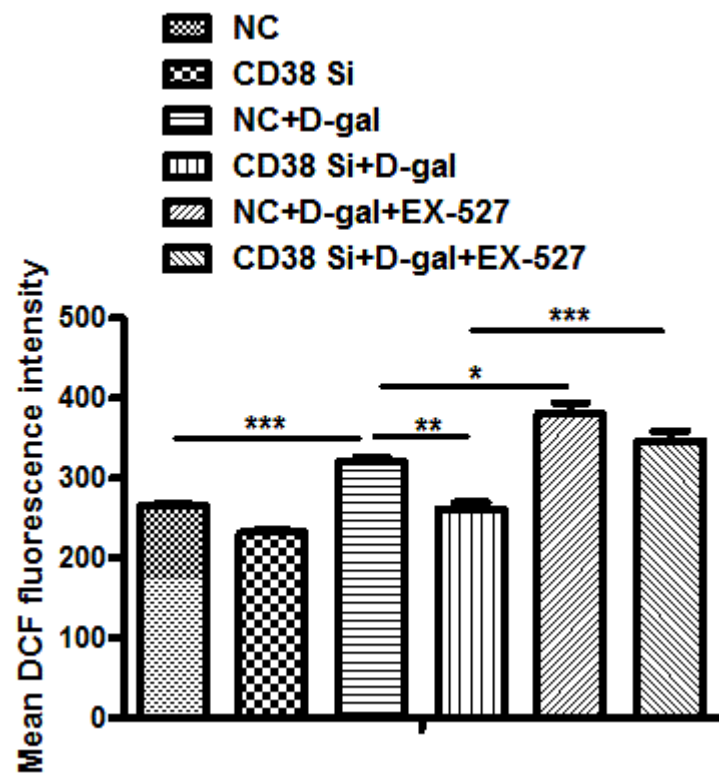

E

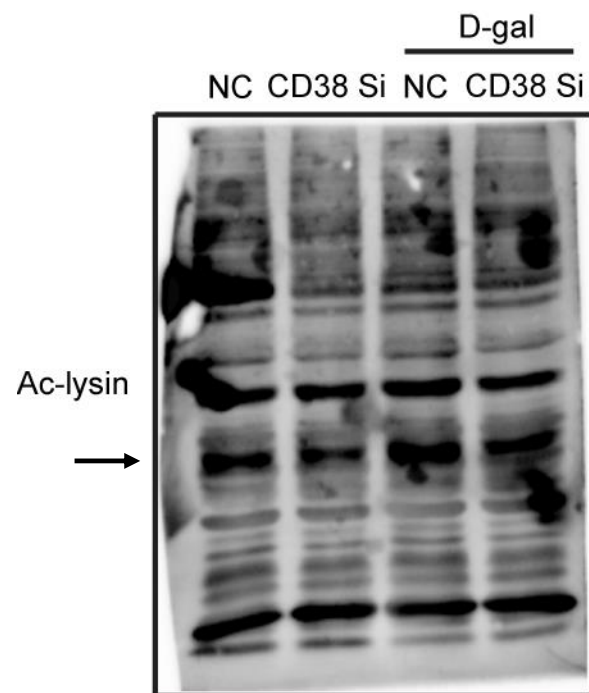

Supplement: FIGURE S1 — Sirt1 was responsible for D-gal induced senescence and oxidative stress and reversed the effects of CD38 knockdown on it. (A) The western blot image of CD38 protein were determined in heart tissue from 1 month and 12 month old mice. (B) The western blot image of Sirt1 protein in H9c2 cells treated with D-gal (10 g/L). (C) SA-β-gal staining in CD38 knockdown H9c2 cells treated with or without with D-gal (10 g/L) or combined with Sirt1 specific inhibitor EX-527 (25 μM). (D) The mean fluorescence intensities of ROS production were quantitatively analyzed in H9c2 cells treated with D-gal (10 g/L) or combined with EX-527 (25 μM). (E) The protein acetylation level of CD38 knockdown H9c2 cells treated with or without with D-gal (10 g/L) was analyzed by western blotting with antibody against acetylated-lysine. Data are shown as mean ± SEM, ∗p < 0.05, ∗∗p < 0.01 and ∗∗∗p < 0.001, n = 3 per group. [file Image_1.pdf]
